# Using Museum Collections to Detect Pathogens

**DOI:** 10.3201/eid1602.090998

**Published:** 2010-02

**Authors:** C. Miguel Pinto, B. Dnate’ Baxter, J. Delton Hanson, Francisca M. Méndez-Harclerode, John R. Suchecki, Mario J. Grijalva, Charles F. Fulhorst, Robert D. Bradley

**Affiliations:** American Museum of Natural History and City University of New York, New York, New York, USA (C.M. Pinto); Texas Tech University, Lubbock, Texas, USA (C.M. Pinto, B.D. Baxter, J.D. Hanson, F.M. Méndez-Harcleorode, J.R. Suchecki, R.D. Bradley); Pontificia Universidad Católica del Ecuador, Quito, Ecuador (C.M. Pinto, M.J. Grijalva); Ohio University, Athens, Ohio, USA (M.J. Grijalva); University of Texas Medical Branch, Galveston, Texas, USA (C.F. Fulhorst).

**Keywords:** Trypanosoma cruzi, Chagas disease, vector-borne infections, natural history museums, museum collections, trypanosomiasis, parasites, letter

**To the Editor:** Natural history museum collections have evolved in recent years to meet the challenges of current and future interdisciplinary scientific studies. Many natural history museums have built tissue collections and made digital information (e.g., photographs, publications, geographic coordinates) freely available on the Internet. These collections provide endless opportunities to conduct studies, including temporal and spatial surveys of emerging and reemerging pathogens (*1*). We report an example of a museum collection being useful in detecting *Trypanosoma cruzi*, the etiologic agent of Chagas disease, in the southern plains woodrat (*Neotoma micropus*) in southern Texas. This finding is of interest in the epidemiology of Chagas disease because the climatic characteristics and demographics of the region are similar to areas in Latin America where Chagas disease is an important zoonotic agent that infects ≈20 million persons (*2*).

Tissue samples from *N. micropus* woodrats archived in the Natural Science Research Laboratory at the Museum of Texas Tech University were evaluated for *T. cruzi* DNA by PCR methods. All samples were originally collected during March 2001–June 2003 from the Chaparral Wildlife Management Area in southern Texas (28º18′N, 99º24′W), 86 km west of the Mexico–US border; some samples had been used previously in other research projects (*3*). Individual rodents were captured with live traps (n = 13) or by excavating middens in which all the nest occupants were collected by hand (n = 146). Animals were later euthanized and tissue samples (heart, kidney, liver, lung, muscle, spleen) were obtained. Tissues were immediately frozen in liquid nitrogen and permanently stored in ultralow-temperature freezers. We extracted 1 DNA sample from each animal’s liver for use in this survey. DNA amplification was performed by using primers specific to *T. cruzi* (TCZ1 and TCZ2) (*4*) under previously standardized conditions and positive controls (*5*). *T. cruzi* DNA was detected in 42 (26.4%) of 159 woodrat samples tested. Males were infected significantly more often (31/82) than females (11/73); sex was not determined for 4 individuals (Score test for a binomial proportion, z = –4.0, p<0.01). Adults had a nonsignificant higher prevalence (24/92) than all other individuals in the remaining age categories combined (14/54) (age was not determined for 13 individuals) (Score test for a binomial proportion, z = –0.02, p = 0.98). Middens that harbored infected individuals (n = 28, mean = 1.8) were not significantly (t = 0.79, df = 84, p = 0.43) more populated than middens that harbored uninfected individuals (n = 58, mean = 1.6).

Woodrats had been shown by using microscopy to be infected by *T. cruzi* and *T. cruzi*–like organisms (*6*); however, no definitive DNA-based confirmation had been performed (*6*,*7*). The results of this research confirm the infection of *N. micropus* woodrats with *T. cruzi* and show a higher prevalence than that reported in previous studies that used other diagnostic methods. These results also point to woodrats as a potentially important reservoir of *T. cruzi* in North America. We hypothesize that the high prevalence is a consequence of the nest-building habits of these rodents. These nests are complexes of dry branches, grasses, and leaves, with a mean diameter of 84 cm, and offer easy access and permanent refuge to triatomine bugs. Woodrats have been found in association with at least 5 triatomine species: *Triatoma gerstaeckeri*, *T.* *lecticularia*, *T. neotomae*, *T. protracta*, and *T. sanguisuga* (*8*). Another factor for consideration is woodrats’ multigenerational midden use, which may enable the permanent occurrence of triatomine colonies and therefore maintain long-term circulation of *T. cruzi*. Whereas recent characterizations of North American strains have included isolates from other mammalian reservoir hosts (*9*), the genotyping of parasites from *N. micropus* woodrats and other woodrats is still to be done.

Despite successful results from tracking pathogens by using material deposited in natural history museum collections (*10*), this practice is not common. We suggest that natural history museum collections be used more frequently, especially for surveying and genotyping *T. cruzi* in mammals, because of the importance of such information in clarifying the epidemiology and the evolutionary history of this pathogen.
